# Assessing the Pharmacotherapy and Clinical Outcomes After Deep Brain Stimulation for Treatment-Refractory Obsessive–Compulsive Disorder: A Case–Cohort Study

**DOI:** 10.3390/jcm13216549

**Published:** 2024-10-31

**Authors:** Joshua Knebel, Robert K. McClure, M. Lindsey Hedgepeth Kennedy

**Affiliations:** 1 Department of Pharmacy Practice, University of the Incarnate Word, San Antonio, TX 78209, USA; 2 Department of Psychiatry, University of North Carolina, Chapel Hill, NC 27599, USA; robert_mcclure@med.unc.edu; 3 Department of Pharmacy, University of North Carolina, Chapel Hill, NC 27599, USA; lindsey_kennedy@med.unc.edu

**Keywords:** pharmacotherapy, deep brain stimulation, obsessive compulsive disorder

## Abstract

**Background:** In the search for effective treatments for refractive obsessive–compulsive disorder (OCD), deep brain stimulation (DBS) serves as an alternative option for those with minimal response to pharmacotherapy. The rarity of reports regarding DBS use for OCD is attributed to the invasive nature of the procedure: placement of electrodes within targeted areas of the brain to provide neuromodulation. This treatment of last resort may decrease functional impairment and pharmacologic complications for a debilitating mental illness. This study compares the pharmacotherapy utilization and treatment outcomes of five treatment-refractory OCD patients after the placement of DBS with those of a matched cohort. **Methods:** This retrospective, single-center, case–cohort study reviewed the electronic medical records of five subjects treated with DBS for treatment-refractory OCD and compared them to a similar treatment-refractory cohort whose OCD was treated without the use of DBS. Control subjects were matched by age, sex, years since diagnosis, number of previous medication class trials, and additional clinical factors. Inclusion criteria were defined as those that are at least eighteen years of age, assigned a primary diagnosis of OCD per the ICD-10 classification, and received DBS treatment for refractory OCD. Exclusion criteria included comorbid psychotic disorders, unstable neurological or coagulation disorder(s), and/or an eating disorder diagnosis. The primary endpoint was the change in the number of psychotropic medications two years after implantation for the DBS cohort and two years after psychiatric decompensation for the comparator cohort. Secondary endpoints included: Y-BOCS (the Yale–Brown Obsessive–Compulsive Scale) changes over time, duration quantity of psychotropic medication classes prescribed, and additional symptomology scale changes. **Results:** Patients receiving DBS were more likely to be on fewer medications and trialed fewer medications after treatment. One out of the five patients was found to be a responder in Y-BOCS scoring after DBS treatment. A reduction in anxiety and depression symptoms was also seen in the HAM-A and HAM-D scales for those that received DBS. **Conclusions**: A reduction in psychiatric medications trialed during therapy was observed, as well as varying reductions in OCD, anxiety, and depression symptomology following DBS. Results from this study indicate that DBS implantation may contribute to a reduction in polypharmacy while displaying DBS’s potential impact on comorbid anxiety and depression symptoms. Given that the small sample size limits generalizability, additional prospective, randomized trials comparing the efficacy of DBS for OCD-specific symptomology and its overall impact on pharmacotherapy are needed in order to further establish the role of DBS as an accepted treatment option for OCD.

## 1. Introduction

Obsessive–compulsive disorder (OCD) is characterized by persistent thoughts (obsessions) and repetitive, ritualistic behaviors (compulsions) that provoke significant subjective distress (at least 1 h per day) and interfere with social and occupational functioning [1]. First-line treatments for OCD include antidepressants and cognitive behavioral therapy (CBT) [2,3,4,5]. The Y-BOCS (The Yale–Brown Obsessive–Compulsive Scale) is commonly used as the main outcome measure to assess this response to treatment in OCD. Non-response is defined as a reduction in <25% in Y-BOCS scores, whereas response is characterized as a 35–50% score reduction [6]. Unfortunately, 40–60% of the OCD patients do not respond to first-line pharmacotherapy or CBT, and despite the availability of several additional treatment approaches for OCD, full remission is quite rare [7,8,9]. In the search for novel methods of treating refractory OCD, many medication therapies are trialed in order to illicit any form of response, resulting in a heterogeneous response to treatment and complex drug regimens being implemented [10,11]. These factors often result in higher levels of patient disability, as documented by the WHO listing OCD among the ten medical conditions associated with worldwide disability due to the greater instances of financial burden, medication nonadherence, and increased side effects encountered by polypharmacy and treatment resistance [12,13]. In the search for novel methods of treating refractory OCD, innovative studies have investigated deep brain stimulation (DBS) as a last resort treatment [14,15].

DBS is a treatment involving the implantation of electrodes into specific regions of the brain with the aim of attenuating altered activity in affected circuits. The technique consists of a pulse generator that is surgically implanted in the chest and an extension cable that runs from it, under the skin of the neck and scalp, to an electrode implanted in the brain. The parameters for the pulse generator are set by clinicians via a computer that communicates with the pulse generator. DBS provides an adjustable and reversible means of neuromodulation with infrequent serious adverse effects. Stimulation targets for DBS in patients with OCD include the ventral anterior limb of the internal capsule, ventral striatum, subthalamic nucleus, and nucleus accumbens [14,15]. Despite the efficacy and tolerability of DBS and its potential to improve the lives of many patients with refractory OCD, evidence for the effectiveness of DBS in OCD remains limited. The efficacy of DBS for OCD still remains exploratory, as only one randomized controlled trial in addition to several case–cohort studies has investigated its impact [16,17,18,19,20,21,22]. Consequently, there is an urgent need for clinical studies examining the effectiveness of DBS for OCD, especially in terms of the pharmacotherapy utilized throughout treatment [2].

The majority of studies on DBS in OCD are case or pilot studies with smaller samples and are often conducted in strict experimental settings. The largest clinical cohort study produced by Denys and colleagues in 2020 suggested both treatment safety and effectiveness of DBS of the ventral anterior limb of the internal capsule for patients with treatment-refractory OCD in a traditional clinical setting [15,22]. In their clinical cohort of 70 patients, they found that the Y-BOCS, HAM-A, and HAM-D scores all decreased significantly during the first 12 months of DBS. Twelve months of DBS resulted in a mean Y-BOCS score decrease of 13.5 points (40% reduction; effect size = 1.5). HAM-A scores decreased by 13.4 points (55%; effect size= 1.4), and HAM-D scores decreased by 11.2 points (54%; effect size = 1.3). At the 12-month follow-up, 36 of the 70 patients were categorized as responders (52%), 12 patients as partial responders (17%), and 22 patients as nonresponders (31%). Adverse events included transient symptoms of hypomania, agitation, impulsivity, and sleeping disorders. These results hint at the possible clinical effect size of DBS but still highlight the need for further long-term analysis of clinical outcomes associated with this experimental treatment as well as changes in medication therapy that accompany potential treatment responses [1,2].

To date, no studies have investigated the long-term prescribing patterns in those treated for refractory OCD with DBS. Additional information regarding the impact that DBS has on pharmacologic interventions in addition to overall OCD symptomology will help provide insight for clinicians about the benefits and risks of DBS therapy as well as the overall roll of pharmacotherapy in treatment-refractory patients. This study seeks to describe the treatment outcomes of five treatment-refractory OCD patients and outline the prescribing patterns associated with their response to DBS therapy in comparison to a similar treatment-refractory cohort being clinically managed without the use of DBS.

## 2. Methods

This retrospective, single-center, case–cohort study reports the findings from 5 patients who received treatment with DBS for treatment-refractory OCD and a matched cohort. Data included all consecutive OCD patients who received DBS at a University of North Carolina Hospital treatment center between January 2009 and May 2021. Participants were treated in the outpatient setting and had a primary diagnosis of OCD according to DSM-4 criteria and were considered to be treatment-refractory by the supervising physician due to the longevity of their illness, corresponding functional impairment, and nonresponse to greater than or equal to two psychotropics. Patients’ pharmacotherapy and general psychiatric care were managed by the same psychiatric team within the University of North Carolina Health System. Patients were seen by this team for several years before being deemed candidates for DBS implantation. Internal records on medication augmentation, electrode manipulation (voltage settings, etc.), Y-BOCS scores, and all other aspects of clinical care were collected at each visit. Both objective and subjective clinical data were collected for at least two years post-implantation. Clinical data after two months were unable to be obtained for one DBS patient due to them succumbing to their illness shortly after implantation occurred. Any additional data not included in these departmental records were retrieved from the patients’ electronic health record. Patient privacy for this small sample size was protected via several research protocols, including de-identification of patient data and independent data analysis conducted by a researcher uninvolved with the clinical care of the patients. Full approval for the study was granted by the University of North Carolina’s Institutional Review Board in December 2021.

In order to identify a comparative clinical cohort that did not receive DBS for their OCD treatment, a health system-wide report of the electronic health record was generated in order to obtain a list of patients being treated primarily for OCD within the UNC health system. Patients were included in the data extraction if they had a primary diagnosis of OCD (ICD-10 code F42 and all subcodes), were between the ages of eighteen and ninety-nine at the time of their initial appointment, the encounter was not a hospital or lab encounter, and if the patient was not a prisoner at any point during treatment. Patients excluded from the extraction included those who had an encounter-level diagnosis for coagulation disorders (ICD-10 code D68 and all subcodes), schizophrenia (ICD-10 code F20 and all subcodes), PTSD (ICD-10 code F43 and all subcodes), bipolar disorder (ICD-10 code F31 and all subcodes), or eating disorder (ICD-10 code F50 and all subcodes). The data extraction yielded 295 patients.

The clinical cohort for comparison with the DBS recipients was further derived from these 295 patients via matching criteria. Patients were matched to the DBS cohort in a stepwise manner (one-to-one) in order of the following criteria: sex, age at onset of refractory symptoms/decompensation, number of baseline psychotropic medications, and overall disease severity. This matching allowed for a cohort of patients with a primary diagnosis of severe OCD, being treated within the same facilities, to be identified.

Clinical records for the identified groups were reviewed for the 2 years prior and 2 years post DBS implantation for the DBS group or date of first psychiatric decompensation, defined as requiring inpatient medical treatment, for the non-DBS group.

Clinical symptomology scales were completed by nurses or physicians trained in the administration of these instruments before DBS implantation surgery, after implantation, and then at subsequent follow-up appointments. Due to the symptomology scales not being a part of the standard screening procedures in our outpatient clinics, symptomology scores were often not incorporated into the care of the comparator, non-DBS group. Symptomology data were included for all patients receiving DBS, as it is readily obtained as a part of system protocols at each visit for those receiving DBS treatment. All of the recorded symptomology data available for the matched cohort are included in the corresponding data tables. Due to this missing symptomology data, comparisons between groups will be focused on pharmacotherapy outcomes, not symptomology outcomes. Data on symptomology outcomes will be presented for the DBS group without comparison to the clinical cohort as a result. Data on adverse events regarding DBS treatment were collected during each visit via patient observation, self-reporting, or questioning. Secondary pharmacotherapy-based endpoints were gathered based upon available practitioner notes, prescription data, and electronic medical records.

Descriptive statistics were used in the analysis of the data. Measures of central tendency (mean, median, mode) and variation (range) were used to report corresponding results. Calculated changes in symptomology and medications utilized were then displayed in the following data tables for direct comparisons.

## 3. Outcome Measures

The primary outcome measure was the change in the number of psychotropic medications two years after implantation for the DBS cohort and two years after psychiatric decompensation for the comparator cohort.

Secondary outcomes included both clinical and pharmacotherapy-based endpoints. Direct clinical endpoints included the change in Y-BOCS scores. Patients were considered to be responders if they had a score decrease of at least 35% and partial responders if they had a Y-BOCS score decrease between 25% and 34%. Patients were considered to be nonresponders if they had a Y-BOCS score decrease of less than 25%. Measures of anxiety and depression symptomatology included the Hamilton Anxiety Rating Scale (HAM-A) and the 17-item Hamilton Depression Rating Scale (HAM-D). Pharmacotherapy endpoints included the total number of medications trialed before treatment, baseline drug class frequencies of before treatment, number of psychiatric medications trialed throughout treatment, and number of medications at the end of treatment. Patients were deemed to have trialed a medication if they had a prescription written for the corresponding medication in their medical records. Medications were defined as coming from a psychiatric drug class if they came from the following medication classes: antipsychotics, selective serotonin reuptake inhibitors, selective norepinephrine reuptake inhibitors, tricyclic antidepressants, mood stabilizers, central nervous system stimulants, sedative hypnotics/sleep aids, or atypical antidepressants.

## 4. Results

From January 2009 to May 2021, a total of five patients received deep brain stimulation for the treatment of their OCD within UNC Health according to the predetermined inclusion/exclusion criteria. A matched comparator cohort was then generated to compare outcomes in treatment. Baseline demographic data for each individual patient can be found in Table 1. Pooled baseline characteristic data for each group can be found in Table 2. Overall, baseline demographics were revealed to be well-matched between groups with an all-male population, a mean age of 44.1 years, ages ranging from 32 to 54 years, average symptom onset at 16.98 years of age, and having had an average duration of illness of 27.28 years before DBS implantation or psychiatric decompensation (Table 2). Baseline symptomology scores for the DBS treatment group included 37.8 for Y-BOCS, 27.0 for HAM-A, and 25.6 for HAM-D (Table 1).

Baseline psychotropic pharmacotherapy, including the number of medications patients were on prior to DBS implantation or psychiatric decompensation (event that distinguished the start of data collection/observation) and the number of previously trialed medications for each individual patient, can be found in Supplementary Table S3. The specific medications that the patients were on at baseline (Supplementary Table S1) can be found in the Supplementary Materials. Additionally, the previously trialed medications obtained from the available electronic medical records are included in Supplementary Table S2. Patients were on an average of 4.0 medications at baseline. All patients were receiving an SSRI and 60% an antipsychotic. The corresponding rates for having trialed fluvoxamine and/or a TCA were 50% and 30%, respectively. On average, patients had trialed 9.7 medications, including 3.63 SSRIs and 1.1 antipsychotics, previously (Table 3).

**Figure 1:**
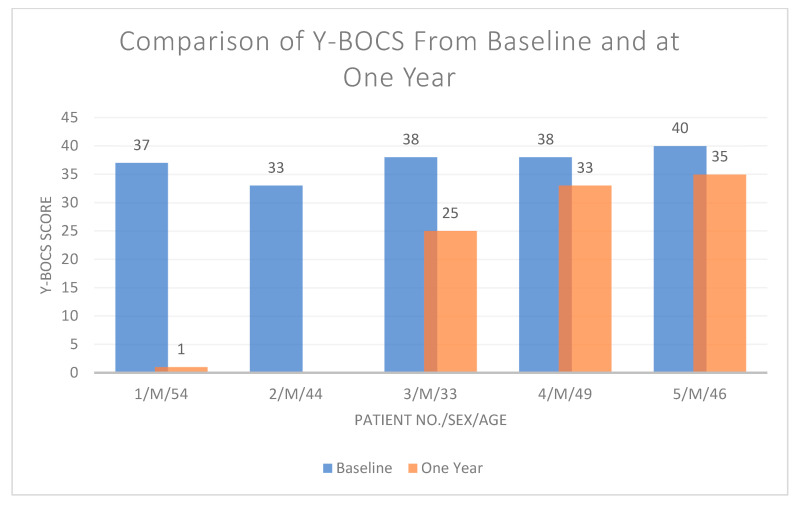
Comparison of Yale–Brown Obsessive–Compulsive Scale (Y-BOCS) at baseline vs. one year after implantation/decompensation.

At the end of the two-year period of treatment/observation, patients had trialed 4.3 new psychiatric medications and were on three medications on average at the end of observation (Table 4). Baseline symptomatology scores for Y-BOCS, HAM-A, and HAM-D are compared to their corresponding scores after one year of DBS treatment in Table 5, Table 6 and Table 7, respectively. The corresponding changes for Y-BOCS (Figure 1), HAM-A (Supplementary Figure S1), and HAM-D (Supplementary Figure S2) are also displayed graphically. One out of the five patients receiving DBS treatment was found to be a responder to treatment (Y-BOCS score > 35%) (Table 5).

## 5. Discussion

The present study explored the treatment outcomes of five patients with treatment-refractory OCD that received deep brain stimulation in comparison to a real-world, matched cohort treated without the use of DBS within the outpatient setting of a major academic health system in the United States. This was accomplished through the generation of a patient cohort comparable to those receiving DBS treatment, as medical records were pulled from the electronic medical record and patients were matched based upon key clinical characteristics. This analysis represents the first of its kind as it followed the pharmacotherapy utilized throughout treatment in addition to therapeutic outcomes such as change in Y-BOCS score.

In the two years of therapy under investigation, DBS patients trialed 5.1 less medications on average and were likely to be on 0.9 less medications at the end of the study period compared to the matched cohort. DBS patients had 1.9 fewer psychiatric prescriptions after treatment, whereas the matched cohort did not see a reduction in the number of their psychiatric medications after treatment. The data can loosely be correlated to the clinical benefits of DBS contributing to patients requiring less pharmacotherapy as a part of their OCD treatment due to its effects on symptomology, etc. Reducing the amount of pill burden, especially in those with severe mental illness, has been linked to improved adherence and less financial burden [19,23]. The side effect profiles of first-line medications and the augmentation agents are also very significant. A reduction in polypharmacy and the corresponding side effect burden could also impact overall mood and how a patient views their disease state, especially in those being treated for OCD whose hallmark symptoms involve obsessions and compulsions [24]. Despite these benefits of reducing the consequences of polypharmacy, these must be compared to the overall symptom control for those with OCD. Patients benefited from a reduction in pharmacotherapy and comorbid anxiety/depression symptomology scores, but it remains unclear if the lack of response in our cohort’s OCD symptoms was also influenced by a reduction in targeted pharmacotherapy or DBS itself.

The number of previously trialed medications, number of baseline medications, and severe Y-BOCS scores display the severity of illness in our patient cohorts. Patients were on an average of 4.4 medications in the DBS group and 3.6 medications in the comparator cohort at baseline. Given that most psychotropic medications work through augmentation of the central nervous system, this leaves patients with refractory disease states prone to CNS depression and other adverse effects as patients trial complex pharmacotherapy regimens in search of symptom relief. SSRIs remained the primarily utilized drug class, with patients having trialed 5.25 medications prior to DBS implantation. Given the documented latency in effect for SSRIs, the duration of illness is often prolonged in OCD and may require the use of agents that offer more immediate anxiolytic effects, such as benzodiazepines, which were being utilized in 60% of patients at baseline. This is more than would be expected, considering that benzodiazepines are not recommended for OCD due to limited efficacy [4,13].

After 1 year of treatment with DBS, one patient out of five was classified as a responder, indicating a response rate of 20%. Obsessive–compulsive symptoms decreased on average by 39% according to Y-BOCS scoring, but this is influenced heavily by the scores of the one patient that responded to treatment. This treatment response rate is lower in comparison to other clinical cohorts and meta-analyses that cite an average Y-BOCS reduction of 47% [14,22]. This lower response rate was most likely influenced by the small sample size and lack of outcome data for one patient that succumbed to their illness during treatment. Of note, one patient had a 34% reduction in symptomology, which is not considered to be a responder based on preset cutoffs, but if they were included in the response category our response rate would rise to 40%, which reflects a response rate similar to other studies. Both anxiety and depression symptoms decreased significantly. Anxiolytic and antidepressant effects were observed in all patients, including non-responders. This reduction in anxiety and depression symptoms is higher than other cohorts that report reductions at approximately 50% [18]. Possible explanations for this trend in a greater reduction in general anxiety and depression include that DBS treatment may be beneficial in treating other psychiatric conditions, the psychotropics used in the treatment of OCD are often used to treat co-morbid depression/anxiety, low rates of comorbid psychiatric diagnoses in the sample, and that the decreased pill burden/polypharmacy may improve overall mood [22,23,24]. Outcomes of DBS when integrated into the clinical care of the cohort, overall, indicate a lower response to OCD symptomology but improvement in comorbid psychiatric illness.

Consideration of DBS and its potential impact for severe OCD in terms of symptomology and pharmacotherapy must also be weighed in comparison to other treatments. Several new augmentation strategies and forms of psychotherapy are emerging for those with treatment-resistant OCD. The effectiveness of exposure and response prevention (ERP) therapy has been documented when combined with traditional CBT [25]. Other invasive procedures, such as transcranial direct current stimulation (tDCS) and vagus nerve stimulation (VNS), remain as alternative options. This study seeks to provide additional outcomes for those receiving DBS and highlights the importance of targeted pharmacotherapy in those with severe OCD [26]. Continuous monitoring of the outcomes of those receiving DBS for OCD will further define its place in the multifactorial approach to OCD treatment [27].

The findings should be interpreted in the context of a small sample size with corresponding strengths and limitations. The study population reflects a population referred to an outpatient clinic within a large, academic medical system. Strengths include the minimization of measurement bias via the same group of clinicians administering the symptom scales. DBS implantation and optimization were completed by the same group of physicians and monitored according to standardized protocols. The comparator cohort was also treated within the same health system, utilizing the same procedures as those that received DBS. Study limitations can be linked back to the study design, as the retrospective nature and small sample size limit the applicability of controlled treatment environments and assessment of global treatment trends. Patients also had psychotherapy and medication adjustments throughout treatment in addition to their DBS setting adjustments. The combination of psychotherapy, pharmacotherapy, and other therapies remains the standard of care, especially in those with severe OCD [27]. Therefore, changes in medication usage and OCD severity cannot necessarily be attributed to DBS alone, and the overall small sample size limits the generalizability of the results. The absence of Y-BOCS, HAM-A, and HAM-D scores limits the ability to compare the two groups in terms of therapeutic efficacy outside of pharmacotherapy comparisons. Given the severity/treatment-refractory diagnosis of OCD in the studied patients, the impact of pharmacotherapy and psychotherapy may be limited, though. The cohorts were also not matched according to ethnicity, which introduces another confounding variable as cultural factors have been shown to influence the expression and perception in the treatment of severe mental illness. Race, spirituality, and country of origin are a few of these cultural factors that could have influenced treatment response [28]. Limited symptomology data for the comparator cohort and missing data for one patient that received DBS treatment also limited comparison of Y-BOCS scores across groups.

## 6. Conclusions

In the present long-term follow-up study comparing the outcomes of patients treated with treatment-refractory OCD to a matched cohort, a reduction in psychiatric pharmacotherapy was observed and a (>35% Y-BOCS reduction) occurred in one of the five patients treated with DBS. Results from this study indicate that DBS implantation may contribute to a reduction in polypharmacy while displaying DBS’s impact on comorbid anxiety and depression symptoms. Given that the small sample size limits generalizability, additional prospective, randomized trials comparing the efficacy of DBS for OCD-specific symptomology and its overall impact on pharmacotherapy are needed in order to further establish the role of DBS as an accepted treatment option for OCD.

## Figures and Tables

**Table 1:** Baseline demographics of DBS and comparative cohort.

DBS Cohort							
Patient No./Sex/Age at DBS Implant	Age at OCD Symptom Onset	Duration of OCD (Before Implantation) in Years	Date of Implantation	Psychiatric Comorbidity	Score		
					Y-BOCS	HAM-A	HAM-D
1/M/54	21	33	15 March 2010	MDD	34	29	29
2/M/44	--	--	31 October 2011	--	38	36	33
3/M/33	10	23	27 August 2012	MDD	40	11	9
4/M/49	5	44	22 April 2013	MDD	40	40	40
5/M/46	19	27	19 January 2016	None	37	19	17
**Comparator Cohort**							
**Patient No./Sex/ (Decomp.)**	**Age at OCD Symptom Onset**	**Duration of OCD (Before Decompensation)**	**Date of Decompensation**	**Psychiatric Comorbidity**	**Score**		
					**Y-BOCS**	**HAM-A**	**HAM-D**
6/M/54	27	27	5 July 2017	GAD, MDD	28	--	--
7/M/45	20	25	19 April 2018	ADHD, MDD	--	--	--
8/M/34	23	11	4 March 2013	MDD, ADD	--	--	--
9/M/50	14	36	20 March 2013	MDD, AUD	31	--	--
10/M/32	17	15	2 November 2016	GAD, OUD	--	--	--

**Table 2:** Average baseline demographics of DBS and comparative cohort.

Baseline Characteristic	DBS (*n* = 5)	Comparative Clinical Cohort (*n* = 5)
Gender (% Male)	5 (100)	5 (100)
Age (Years)	45.2	43.0
Age Range	33–54	32–54
Age of OCD Symptom Onset	13.75	20.2
Duration of Illness Before Intervention (Years)	31.75	22.8
Y-BOCS	37.8	--
HAM-A	27.0	--
HAM-D	25.6	--

**Table 3:** Average baseline medication history before treatment.

Baseline Characteristic	DBS (*n* = 5)	Comparator (*n* = 5)
Baseline Psychotropic Meds	4.4	3.6
Receiving SSRIs/SNRIs (%)	5/5 (100)	5/5 (100)
Receiving Antipsychotic (%)	3/5 (60)	3/5 (60)
Trialed Fluvoxamine	3/5 (60)	2/5 (40)
Trialed Clomipramine/TCA	3/5 (60)	0/5 (0)
Previously Trialed Psychotropic Meds	13.0	6.4
Average Number of Trialed SSRIs	5.25	2
Average Number of Trialed Antipsychotics	1	1.2

**Table 4:** Average change in psychiatric medications during treatment.

	DBS	Comparator Cohort
Baseline Psychiatric Medications	4.4	3.6
Psychiatric Medications at 2 Years	2.5	3.4
Psychiatric Medications Trialed Over 2 Years	1.5	6.6

**Table 5:** Baseline Y-BOCS symptomology scores vs. one year after implantation for those treated with DBS.

Patient No./Sex/Age	Baseline Y-BOCS	1-Year Y-BOCS	% Reduction	Y-BOCS Responders (>35% Improvement)
1/M/54	37	1	97%	Y
2/M/44	33	--	--	N
3/M/33	38	25	34%	N
4/M/49	38	33	13%	N
5/M/46	40	35	13%	N

**Table 6:** Baseline HAM-A symptomology scores vs. one year after implantation for those treated with DBS.

Patient No./Sex/Age	Baseline HAM-A	1-Year HAM-A	% Reduction
1/M/54	37	2	95%
2/M/44	33	--	--
3/M/33	15	0	100%
4/M/49	37	15	59%
5/M/46	24	0	100%

**Table 7:** Baseline HAM-D symptomology scores vs. one year after implantation for those treated with DBS.

Patient No./Sex/Age	Baseline HAM-D	1-Year HAM-D	% Reduction
1/M/54	35	0	100%
2/M/44	35	--	--
3/M/33	13	2	84%
4/M/49	42	11	74%
5/M/46	27	2	93%

## Data Availability

The data presented in this study are available on request from the corresponding author.

## Supplementary Materials

The following supporting information can be downloaded at: https://www.mdpi.com/article/10.3390/jcm13216549/s1, Table S1: Baseline Psychiatric Medications for All Patients before Implantation or Psychiatric Decompensation; Table S2: Previously Trialed Psychotropic Medications for Both Cohorts Before Analyzing Changes in Pharmacotherapy; Table S3: Baseline Pharmacotherapy Before Treatment by Patient; Table S4. Change in Psychiatric Medications During Treatment by Patient; Figure S1. Comparison of Hamilton Anxiety Scale (HAM-A) at Baseline vs. One Year after Implantation/Decompensation; Figure S2. Comparison of Hamilton Scale for Depression (HAM-D) at Baseline vs. One Year after Implantation/Decompensation.
