# Supplementary material for: Assessing the Pharmacotherapy and Clinical Outcomes After Deep Brain Stimulation for Treatment-Refractory Obsessive–Compulsive Disorder: A Case–Cohort Study
Source: J Clin Med. 2024 Oct 31;13(21):6549. doi: 10.3390/jcm13216549 (PMC11546672; doi:10.3390/jcm13216549)

Supplementary Materials

Table S1: Baseline Psychiatric Medications for All Patients before Implantation or Psychiatric Decompensation

Pharmacotherapy Data Patient	
1/M/54	Risperidone, Paroxetine, Clomipramine, Clonazepam, Mirtazapine
2/M/44	Fluvoxamine, Olanzapine, Valproic Acid, Eszopiclone
3/M/33	Fluoxetine, Ativan, Risperdal Consta, Amitriptyline
4/M/49	Sertraline, Clomipramine, Zolpidem, Trazadone, Vistaril, Clonazepam, Buspirone
5/M/46	Clonazepam, Sertraline
6/M/54	Clonazepam, Furazepam, Zaleplon, Sertraline, Escitalopram, Quetiapine
7/M/45	Xanax, Atomoxetine, Fluoxetine, Aripiprazole
8/M/34	Clonazepam, Lithium, Fluoxetine, Clozapine
9/M/50	Fluoxetine, Lorazepam, Trazodone
10/M/32	Sertraline

Table S2: Previously Trialed Psychotropic Medications for Both Cohorts Before Analyzing Changes in Pharmacotherapy

Pharmacotherapy Data Patient	
1/M/54	Clonazepam, Celexa, Prozac, Wellbutrin, Venlafaxine, Duloxetine, Lamotrigine
2/M/44	--
3/M/33	Lithium, Valproic Acid, Luvox
4/M/49	Lexapro, Ativan, Remeron, Abilify, Xanax, Prozac, Effexor, Luvox, Citalopram, Cymbalta, Wellbutrin
5/M/46	Anafranil, Imipramine, Celexa, Paxil, Buspar, Lithium, Risperdal, Prozac
6/M/54	Escitalopram, Hydroxyzine
7/M/45	Geodon, Strattera, Doxepin, Methylphenidate, Fluvoxamine, Xanax, Aripiprazole, Fluoxetine, Ritalin
8/M/34	Clonazepam, Lithium, Ritalin, Asenapine, Paroxetine, Aripiprazole, Risperdal, Haloperidol, Venlafaxine, Valproic Acid
9/M/50	Trazodone, Buspirone, Fluvoxamine, Lorazepam
10/M/32	Paroxetine

Table S3. Baseline Pharmacotherapy Before Treatment by Patient

DBS		
Patient No./Sex/Age at DBS Implant	# of Baseline Psych Meds	# of Previously Trialed Psych Meds
1/M/54	5	11
2/M/44	4	--
3/M/33	4	12
4/M/49	7	19
5/M/46	2	10
Comparator		

Patient No./Sex/ (Decomp.)	# of Baseline Psych Meds	# of Previously Trialed Psych Meds
6/M/54	6	6
7/M/45	4	8
8/M/34	4	12
9/M/50	3	5
10/M/32	1	1

Table S4. Change in Psychiatric Medications During Treatment by Patient

DBS Cohort			
Patient No./Sex/Age at DBS Implant	Baseline Psychiatric Medications at Implantation	Number of Psychiatric Medications at end of 2 years	Number New of Psychiatric Medications Trialed
1/M/54	4	2	2
2/M/44	4	--	--
3/M/33	2	1	0
4/M/49	5	5	1
5/M/46	1	2	3
Comparator Cohort			
Patient No./Sex/ Age (Decomp.)	Baseline Psych Medications at Decompensation Start	Number of Psychiatric Meds at end of 2 years	Number New of Psych Meds Trialed
6/M/54	6	5	13
7/M/45	4	3	6
8/M/34	4	2	1
9/M/50	3	3	8
10/M/32	1	4	5

Figure S1. Comparison of Hamilton Anxiety Scale (HAM-A) at Baseline vs. One Year after Implantation/Decompensation

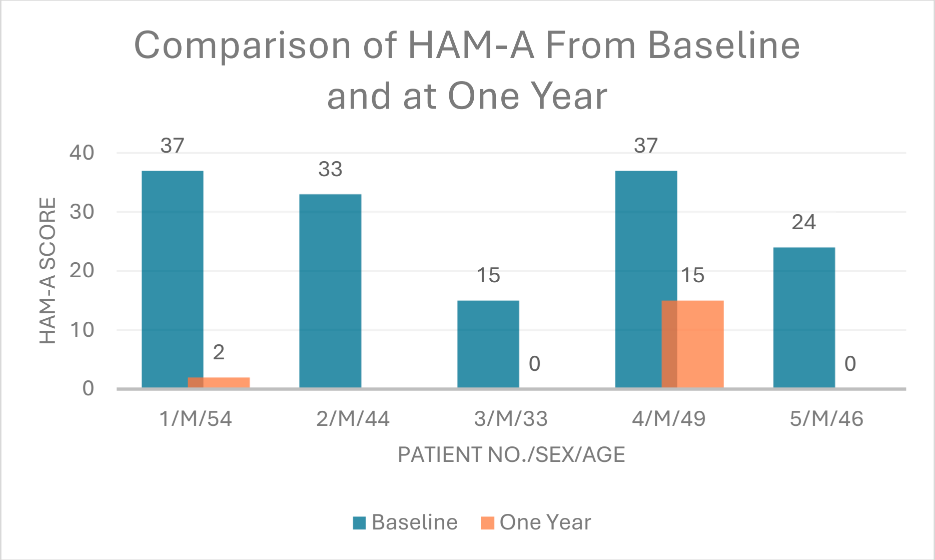

Figure S2. Comparison of Hamilton Scale for Depression (HAM-D) at Baseline vs. One Year after Implantation/Decompensation

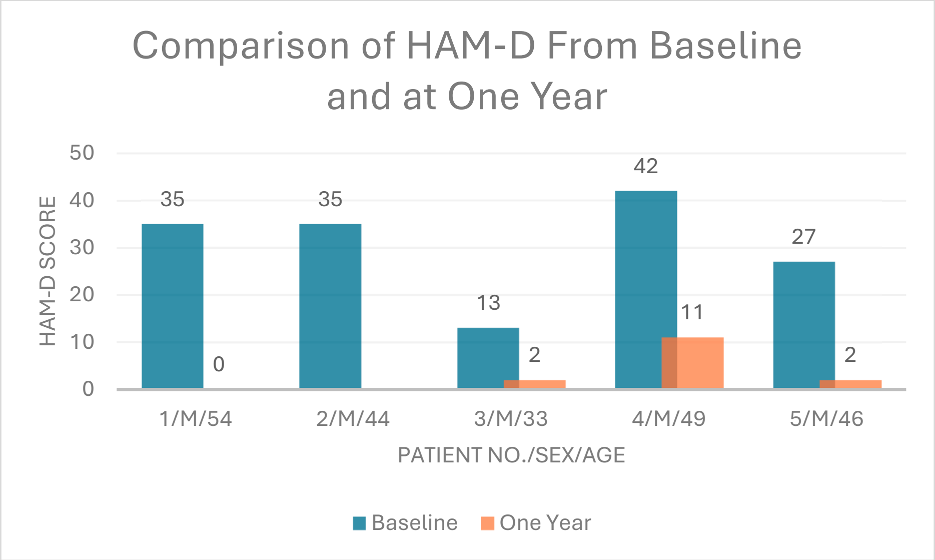

Supplement: Supplementary file 1 [file jcm-13-06549-s001.zip › jcm-3267910-supplementary.pdf]
